# Hypodermic Injections for the Obliteration of Varicose Veins

**Published:** 1865-06

**Authors:** E. Andrews

**Affiliations:** Professor of Surgery in Chicago Medical College


### ARTICLE XIX.

### HYPODERMIC INJECTIONS FOR THE OBLITERATION OF VARICOSE VEINS. A Clinical Lecture.

By E. ANDREWS, M.D., Prof. of Surgery in Chicago Medical College.

The question of the best method of obliterating varicose veins is still asked with great interest among practical men, because there is great discrepancy of opinion among the standard works on the subject.

The three methods of ligature, caustic, and hypodermic injection, are the ones between which we usually have to choose.

Of these, the ligature is doubtless the most dangerous. Owing to the irregularity of the veins, and their numerous branches, it is practically impossible to ligate extensively without occasionally transfixing some venous trunk or branch, and passing the needle and ligature through it instead of under it. This makes a fistulous channel at once from the external air to the interior of the vein, and indeed carries a small quantity of the air enclosed among the fibres of the ligature into the vessel, both of which circumstances favor decomposition, erysipelas, and pyæmic phlebitis. On this account occasional deaths follow the operation, and surgeons have a well-founded dread of it.

To avoid this, some prefer the use of caustic. Prof. Gross advises that a mixture of equal parts of caustic potash and quicklime be made into a paste with alcohol, and applied in small spots over the veins, leaving it on from ten to twenty minutes, until an eschar is formed, reaching to the vein. Gross thinks this course to be quite safe. It has the advantage of not laying the vein open to the air, and though ulcers formed by the fall of eschars may sometimes provoke erysipelas and pyæmia, as even a pin scratch may do in persons strongly predisposed to it, yet it is a rare occurrence, and the procedure must be reckoned as safe as any minor operation in surgery. The practical inconveniences of the method are the following: 1st. It often happens that some of the caustic patches fail to reach the vessel, and after weeks of delay a repetition of the process is necessary. 2d. The issues formed by the caustic require several weeks to heal. 3d. The caustic plan is not applicable to erectile tumors, especially those on the face and neck. It is most successful in common varicose veins of the legs.

The third method consists in the introduction of a solution of perchloride or persulphate of iron into the vessels by the means of a fine-pointed hypodermic syringe. To accomplish this, the limb is ligated as for venesection, in order to distend the vessels. The point of the syringe is inserted at various places, and at each spot three or four drops of the liquor ferri perchloridi, or of Mansol's solution of the persulphate are inserted.

the clots, and were effectually cured without a particle of suppuration.

CASE III. An infant, four days old. This child was born with an erectile tumor upon the back of the neck, which speedily grew to the size of an egg, and threatened to increase still more. It might, from its location, have been easily excised, but, from its depth and breadth of base, I feared more hemorrhage than would be safe in a new-born infant. I therefore inserted ten drops of mur. tinct. of iron with the hypodermic syringe, passing it through the tumor in various directions. This at once converted it into a hard, knobby mass, with most of the veins well obliterated. After a few weeks, however, some of the remoter corners of the tumor proved still vascular, and required two additional injections. The result of the repetition was to make a small abscess in the tumor which discharged, after which the whole mass shrivelled away to a small fleshy knob.

CASE IV. A young man, aged 18 years. He had several deep nævi involving in the tumors, the whole thickness of the left cheek, and part of one lip, and making a tumor 2½ inches long on the side of his neck. I inserted about twenty drops of the mur. tinct. of iron into each of these tumors, without any anæsthetic. They were converted at once into hard instead of soft swellings. Some inflammation followed, but no suppuration, and the masses began to shrink away, when the patient left for home, apparently cured.

I believe this method to be the best known for vascular tumors, and much safer than the ligature, in the common varicosities of the leg. It has promptness and certainty in its favor also. I do not believe that either this operation, or the use of the caustic, nor any other operation in surgery, however trivial, can be made perfectly safe from pyæmic phlebitis, if indiscriminately practiced upon patients in a decidedly aplastic and erysipelatous diathesis. When patients are in such a state of the general system, any scratch or pimple may give origin to fatal erysipelas or pyæmia.

But it should be borne in mind that this matter is very much

The blood at the place of insertion is instantly formed into a hard small clot. The vein contracts upon it and is from that moment obliterated. The puncture, being extremely minute, admits no air, and no fistula is established. The clot seems to be imprisoned by a plug of plastic lymph on either side of it, and is usually absorbed without any suppuration, leaving the patient cured. In erectile tumors this is by far the best of the three methods.

The following cases may serve to illustrate the subject:—

Case I. A woman, aged about 50 years. She had a large erectile tumor, of about three inches in diameter, upon the top of the head. It was arterial rather than venous, as was shown by its distinct pulsations. Part of the thickness of the skull was absorbed beneath it, giving an excavated feeling as the finger was pressed under the edge of the base. As it was rapidly growing, it became necessary to attend to it at all hazards. I first tied all the arterial trunks leading to it, but found that the collateral circulation still kept up the tumor. I therefore took a hypodermic syringe, and inserting its pipe in various directions in the tumor, left within it fifteen drops of the liquor ferri perchloridi. The result was an instantaneous knotty hardness wherever the solution was lodged. After a week, I began to fear that the first injection would not be sufficient, and as the patient began to be in great haste to return home, I made an effectual end of the disease by inserting about thirty drops more. The result of this haste in the treatment was to create a smart inflammation, followed by an abscess in the tumor, which discharged a mixture of pus and black clots, after which the whole mass shrivelled away and was cured.

Case II. A laborer, aged about 35 years, troubled with varicose veins, and an ulcer on the right leg. In this instance I determined to test both the caustic and the injections. I therefore applied the caustic over part of the veins, and inserted the perchloride of iron in solution into the others. The caustic was left on twenty minutes, and was measurably successful in places, but in certain portions failed to obliterate the vessels. The veins which were treated by the syringe, shrunk at once upon under our control. If the patients have their constitutions well prepared beforehand, by keeping them in a current of perfectly pure out-door air for a few days, both night and day, and by giving freely the tincture of iron, and if the same management be kept up during the cure, erysipelas and pyæmia will be almost an unknown accident. But, if they sleep in crowded houses, or in small rooms with closed windows, from the fear of colds, then these surgical scourges will occasionally take their victims from every class of injuries and operations, and no skill can wholly avert the consequences of the folly in breathing impure air.

